# Impairment in Right Ventricular-Pulmonary Arterial Coupling in Overweight and Obesity

**DOI:** 10.3390/jcm13123389

**Published:** 2024-06-10

**Authors:** Athina Goliopoulou, Evangelos Oikonomou, Panagiotis Theofilis, Vasiliki Tsigkou, George Makavos, Islam Kourampi, Maria Katsioupa, Vaios-Dionysios Antoniou, Ignatios Ikonomidis, Vaia Lambadiari, Aikaterini Tsatsaragkou, Savvas Sarantos, George E. Zakynthinos, Manolis Vavuranakis, Gerasimos Siasos

**Affiliations:** 1 Third Department of Cardiology, Thoracic Diseases General Hospital Sotiria, Medical School, National and Kapodistrian University of Athens, 11527 Athens, Greece; agoliopoulou@gmail.com (A.G.); bikytsigkoy@yahoo.gr (V.T.); gmakavos@hotmail.com (G.M.); mkatsioupa@gmail.com (M.K.); antoniouvaios@gmail.com (V.-D.A.); aiktsatsaragou@hotmail.com (A.T.); savvas.sarantos@yahoo.com (S.S.); gzakynthinos2@gmail.com (G.E.Z.); vavouran@otenet.gr (M.V.); ger_sias@hotmail.com (G.S.); 2 First Department of Cardiology, Hippokration General Hospital, Medical School, National and Kapodistrian University of Athens, 11527 Athens, Greece; panos.theofilis@hotmail.com; 3 Second Department of Internal Medicine, Attikon University Hospital, Medical School, National and Kapodistrian University of Athens, 12462 Athens, Greece; ignoik@gmail.com (I.I.); vlambad@otenet.gr (V.L.); 4 Cardiovascular Division, Harvard Medical School, Brigham and Women’s Hospital, Boston, MA 02115, USA

**Keywords:** obesity, right ventricular-arterial coupling, heart failure

## Abstract

**Background:** The association of obesity with right ventricular function and the interplay between right heart and pulmonary circulation is incompletely understood. We evaluate the role of obesity as a determinant of right ventricular-pulmonary artery coupling (RVAC). **Methods:** We retrospectively studied consecutive subjects without overt cardiovascular or pulmonary disease. Subjects were stratified according to body mass index (BMI) as normal weight, overweight, or obese. A transthoracic echocardiographic study was used to assess left and right heart functional and structural parameters. RVAC was assessed using the ratio of peak systolic velocity of the tricuspid annulus to pulmonary artery systolic pressure (PASP). **Results:** A total of 145 subjects were enrolled with diabetes mellitus incidence higher in obese. There was no difference in left ventricular global longitudinal strain and in PASP or markers of right ventricular systolic function based on BMI. RVAC was significantly lower in the presence of obesity (normal weight: 0.52 (0.19) cm·(sec·mmHg)^−1^ vs. overweight: 0.47 (0.16) cm·(sec·mmHg)^−1^ vs. obese: 0.43 (0.14) cm·(sec·mmHg)^−1^, *p* = 0.03), even after adjustment for confounders (β: −0.085, 95% confidence interval: −0.163, −0.009, *p* = 0.029). **Conclusions:** Our findings highlight the relationship between metabolic impairment and RVAC, suggesting additional mechanisms for heart failure development observed in obese subjects.

## 1. Introduction

The increasing prevalence of obesity, regardless of geographical boundaries, is emerging as a worldwide healthcare challenge, inevitably closely associated with multifaceted metabolic and cardiovascular complications [1]. This epidemic not only poses a significant burden on healthcare systems but also threatens the quality of life and longevity of affected individuals [2].

Beyond known correlations with metabolic syndrome, diabetes mellitus, and coronary artery disease, obesity’s intricate interplay with the cardiovascular system may extend to heart failure, especially heart failure with preserved ejection fraction (HFpEF) and right heart failure [3]. Obesity significantly influences the likelihood that a symptomatic patient suffers from HFpEF [4] and is correlated with atrial fibrillation development, a common and serious cardiac arrhythmia [5]. Moreover, metabolic-associated fatty liver disease (MAFLD), which frequently accompanies obesity, is implicated in the incidence of left ventricular (LV) diastolic function impairment, further complicating the cardiovascular health of obese individuals [6]. The detrimental impact of obesity extends to worse invasive hemodynamic measurements of right ventricular function in patients undergoing right heart catheterization, highlighting the pervasive effects of excess body weight on cardiac performance [7]. Regarding the underlined mechanisms linking obesity to the impairment of right or left heart function parameters, it can be noted that toxic effects from a pro-inflammatory milieu and cytokines impact on myocardial cells, and endothelial dysfunction, reduced right heart preload reserve, and microvascular impairment are observed [5].

Despite these well-documented associations, the role of obesity in right ventricular function and the interplay between the right heart and pulmonary circulation in subjects without existing cardiovascular and pulmonary diseases remains less well studied. This gap in knowledge is particularly significant given the rising prevalence of obesity and its potential to exacerbate undiagnosed or subclinical cardiac conditions [8].

Therefore, in this study, we aim to examine how obesity is associated with right heart ventriculoarterial coupling (RVAC). By investigating the mechanisms and impacts of obesity on RVAC, we intend to shed light on the broader associations of obesity with cardiovascular health and to provide insights that may inform future therapeutic strategies and interventions.

## 2. Methods

### 2.1. Study Design

This is a retrospective, cross-sectional study conducted in the “Sotiria” General Hospital for Chest Diseases from August 2021 to November 2023. In this analysis, we included consecutive subjects without known overt cardiovascular disease who visited the outpatient echocardiographic department. Individuals with a history of cardiovascular disease, current arrhythmia, or poorly controlled hypertension for at least 6 months were excluded to ensure a focus on subjects without significant pre-existing cardiovascular conditions. For the purpose of this study, subjects with chronic obstructive pulmonary disease (COPD) were also excluded, as COPD can independently affect right ventricular function and confound the results. Standard demographic and clinical characteristics were collected for each participant, including age, sex, body mass index (BMI), smoking status, and relevant medical history. To ensure data integrity and reliability, individuals lacking adequate echocardiographic data for RVAC were excluded from the final analysis.

The study’s primary hypothesis was a potential association between obesity, right ventricular performance, and RVAC. Specifically, we aimed to determine whether increased BMI and other markers of obesity are correlated with impaired right ventricular function and altered RVAC, independent of other confounding factors.

The study adhered to the STROBE (Strengthening the Reporting of Observational Studies in Epidemiology) checklist [9], and to the principles outlined in the Declaration of Helsinki (1989). All individuals were informed about the aims of the study and provided written informed consent.

### 2.2. Clinical and Anthropometric Measurements

Baseline demographic and clinical characteristics were recorded in all subjects. Weight and height measurements were conducted in accordance with standard protocols, and body mass index (BMI) was calculated as weight in kilograms divided by height in meters squared (kg/m^2^). Normal weight, overweight, and obese subjects were defined as BMI < 25 kg/m^2^, 25–29.9 kg/m^2^, and >29.9 kg/m^2^, respectively. Body surface area (BSA) was calculated according to Mosteller’s equation (0.20247 × weight^0.425^ × height^0.725^) in m^2^ [10].

Subjects were classified as having hypertension if they were undergoing treatment for arterial hypertension or if the average of three consecutive office blood pressure measurements exceeded 140/90, as per the guidelines of the European Society of Hypertension [11]. Diabetes mellitus status was determined based on specific treatment or according to the criteria set by the European Association for the Study of Diabetes (EASD)/European Society of Cardiology (ESC), which includes a Fasting Plasma Glucose level ≥ 126 mg/dL or a glycated hemoglobin level ≥ 6.5% [12]. Hyperlipidemia was identified in subjects either already receiving hypolipidemic therapy or requiring lipid-lowering therapy based on low-density lipoprotein-cholesterol levels and CV SCORE-2 [13,14].

### 2.3. Echocardiography

All subjects underwent standard transthoracic echocardiography, conducted using the Philips EPIQ CVx ultrasound system (manufactured by Philips, Andover, MA, USA) by the same expert operator in a dimly lit room. The images were analyzed using the Philips Q station 3.9 ultrasound software. A full echocardiography protocol was performed which included all standard views according to the guidelines of the American Society of Echocardiography [15]. The parasternal long-axis view was utilized for a two-dimensional echocardiographic examination of the LV. Measurements were taken for LV end-systolic (LVESD) and -diastolic dimensions (LVEDD), left atrial diameter (LAD), as well as thicknesses of the posterior wall (PW) and interventricular septum (IVS). The mean values were obtained from three consecutive cardiac cycles for individuals with sinus rhythm [15]. Subsequently, LA diameter, LVEDD, and LVESD were indexed to BSA. LV mass (LVM) was calculated with the method of Devereux et al. [16], namely LVM = 0.8 × (1.04 × ((LVEDD (in cm) + IVS (in cm) + PW (in cm))^3^ − LVEDD (in cm) ^3^) + 0.6 was indexed to BSA. LV ejection fraction (LV EF) was estimated with biplane Simpson’s method [15]. The Automated Cardiac Motion Quantification A.I. was utilized for analysis in apical views of the four, three, and two chambers. Following manual adjustments, it facilitated a deformation assessment using 2D speckle tracking technology. This advanced imaging technique allows for the precise tracking of myocardial motion throughout the cardiac cycle, enabling the calculation of left ventricular (LV) Global Longitudinal Strain (GLS). LV GLS is a sensitive marker of myocardial function that quantifies the percentage of deformation of the LV myocardium during systole, providing valuable insights into subtle changes in cardiac performance that may not be evident with traditional measures of ejection fraction. In addition to assessing myocardial deformation, the ratio of trans-mitral inflow velocity to mitral annular e’ diastolic velocity (E/e’) was estimated as a surrogate measure of LV end-diastolic pressure. The E/e’ ratio is a widely recognized echocardiographic parameter used to estimate left ventricular filling pressures, offering an indirect but reliable indication of diastolic function and potential diastolic dysfunction [15]. This ratio is calculated by dividing the peak velocity of early diastolic mitral inflow (E) by the peak velocity of early diastolic mitral annular motion (e’), with higher values suggesting elevated filling pressures and impaired diastolic function.

In the right ventricle-focused apical four-chamber view, we measured the maximum transverse diameter in the basal third of the right ventricle at end-diastole (RVD1_basal_) [15]. Pulmonary Arterial Systolic Pressure (PASP) was estimated by applying the modified Bernoulli equation to the tricuspid regurgitant jet velocity [15]. Additionally, the estimated right atrial pressure was incorporated based on the diameter and collapsibility of the inferior vena cava [15]. Right ventricular systolic performance was estimated using tricuspid annular plane systolic excursion (TAPSE) and using peak systolic velocity of the tricuspid annulus (SRV) [15], while the ratio of SRV-to-PASP was used as a surrogate of right ventriculoarterial coupling (RVAC) [17].

### 2.4. Statistical Analysis

The normality of distribution was examined using the Kolmogorov–Smirnov test alongside visual inspection of P-P plots, ensuring a robust assessment of whether the continuous variables followed a normal distribution. Continuous variables were described as mean (standard deviation). A one-way analysis of variance (ANOVA) was performed to examine for intergroup differences as appropriate. Following the identification of significant differences with ANOVA, post hoc analysis was conducted using the Scheffe correction. Categorical variables were presented as percentages. Differences in categorical variables among groups were evaluated using the chi-square test following the construction of contingency tables, after correction for multiple comparisons. Linear regression analysis was applied to assess the association between increased BMI and RVAC independently of other confounders that found significance in univariate analysis. This regression model allowed us to control potential confounding variables and isolate the effect of BMI on RVAC. All analyses were conducted using two-sided hypotheses, and statistical significance was defined as *p* < 0.05. Statistical calculations were performed in SPSS software (version 27.0; SPSS Inc., Chicago, IL, USA).

## 3. Results

### 3.1. Characteristics of the Study Population

The study population consisted of 145 individuals, whose characteristics according to BMI categories are presented in Table 1. The mean age of the participants was 60.3 (17.0) years, with 53.1% of the subjects being female. The mean BMI was 27.1 (5.4) kg/m^2^, with 40.0% being of normal weight, 36.6% overweight, and 23.4% obese. Concerning cardiovascular risk factors, a history of hypertension, diabetes mellitus, and dyslipidemia was reported by 57.0%, 17.0%, and 36.2% of the subjects, respectively.

When the participants were characterized according to BMI, diabetes mellitus was met at increasing frequencies more frequently in overweight and obese subjects (normal weight: 5.4% vs. overweight: 10.0% vs. obese: 40.7%, *p* < 0.001). However, no major differences were detected concerning other demographic and clinical characteristics according to BMI categories (Table 1). Indeed, between normal weight, overweight or obese, there was no difference in age (57.7 (18.6) years vs. 61.6 (15.3) years vs. 62.9 (16.5) years, *p* = 0.29), male sex prevalence (43.1% vs. 52.8% vs. 44.1%, *p* = 0.55) or hypertension history (50.0% vs. 63.6% vs. 59.3%, *p* = 0.49).

### 3.2. Body Mass Index and Indices of Left Atrial and Ventricular Function

The association between BMI categories and the echocardiographic indices of left cardiac chambers’ structure and function is depicted in Table 2. There was no difference in LV GLS across the BMI categories (normal weight: −20.1 (3.4) % vs. overweight: −20.3 (3.5) % vs. obese: 19.5 (4.0) %, *p* = 0.81). Interestingly, subjects with normal BMI had higher indexed LVEDD (25.7 (2.9) mm/m^2^ vs. 24.2 (2.5) mm/m^2^ vs. 22.2 (2.2) mm/m^2^, *p* < 0.001) and indexed LVESD (16.6 (2.6) mm/m^2^ vs. 16.4 (2.5) mm/m^2^ vs. 15.3 (1.9) mm/m^2^, *p* = 0.016) compared to overweight and obese. PWT was also considerably elevated, especially in obese subjects. Normal weight subjects had an increased LAD indexed for BSA when compared to obese individuals (normal weight: 20.8 (4.2) mm/m^2^ vs. overweight: 20.0 (3.3) mm/m^2^ vs. obese: 18.2 (2.7) mm/m^2^, *p* = 0.06). Last but not least, there was a trend towards high E/e’ in overweight and obese subjects (normal weight: 7.6 (2.7) vs. overweight: 8.6 (3.3) vs. obese: 9.7 (6.0), *p* = 0.056).

### 3.3. Association of Obesity with Right Ventricular Function and RVAC

**Figure 1:**
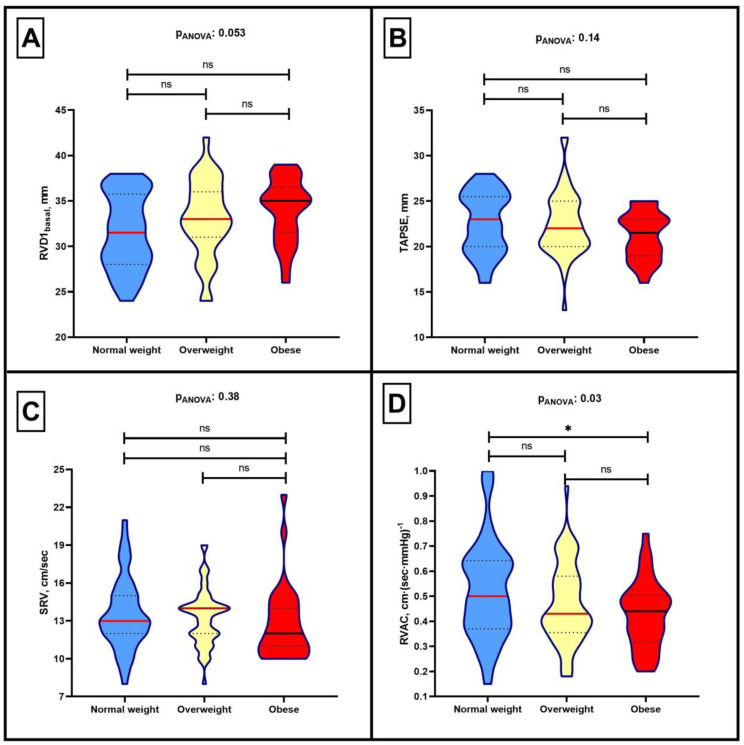
Violin plots demonstrating the differences according to body mass index categories in (**A**) maximum transverse diameter in the basal third of the right ventricle at end-diastole (RVD1), (**B**) tricuspid annular plane systolic excursion (TAPSE), (**C**) peak systolic velocity of the tricuspid annulus (SRV), and (**D**) right ventricular-arterial coupling (RVAC). *: signifies *p* < 0.05, ns = not significant.

**Figure 2:**
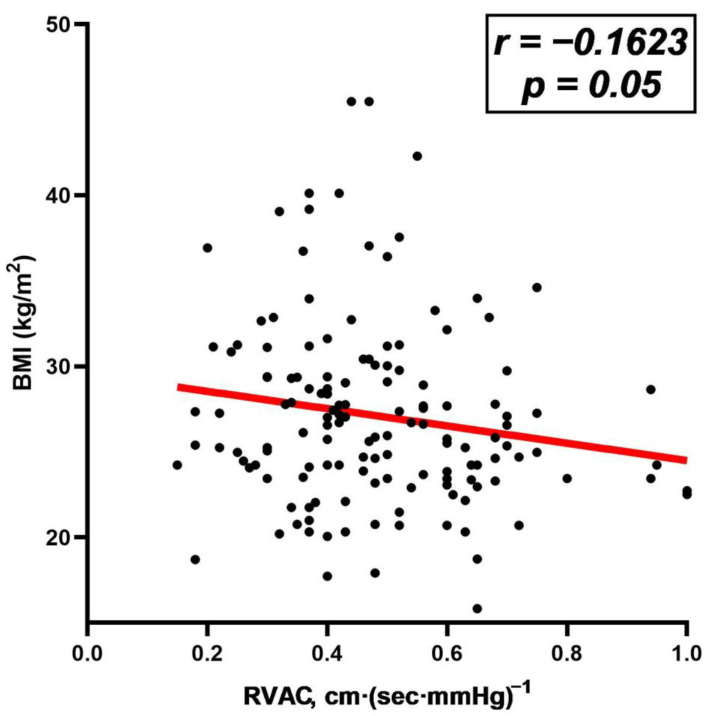
Scatter plot demonstrating the correlation between body mass index (BMI) and right ventricular-arterial coupling (RVAC). Dots are values for individual data point, red line is a trend line showing the mathematically best fit to the data.

We next examined the association between BMI categories and the echocardiographic indices of right ventricular function and RVAC (Supplementary Table S1). RVD1_basal_ exhibited a stepwise increase according to BMI categories (normal weight: 31.6 (4.1) mm vs. overweight: 33.1 (4.3) mm vs. obese: 34.0 (3.5) mm, *p* = 0.053), (Figure 1, Panel A). However, there were no differences in PASP (normal weight: 28 (8) mmHg vs. overweight: 30 (8) mmHg vs. obese: 32 (9) mmHg, *p* = 0.14) or markers of right ventricular systolic function based on BMI (for TAPSE: normal weight: 22.6 (3.4) mm vs. overweight: 22.4 (3.5) mm vs. obese: 21.2 (2.6), *p* = 0.14) and (for SRV: normal weight: 13.5 (2.8) cm/sec vs. overweight: 13.2 (2.3) cm/sec vs. obese: 12.7 (2.9), *p* = 0.38) (Figure 1, Panels B–D). When examining the differences in RVAC, we noted a stepwise decrease across the BMI groups (normal weight: 0.52 (0.19) cm·(sec·mmHg)^−1^ vs. overweight: 0.47 (0.16) cm·(sec·mmHg)^−1^ vs. obese: 0.43 (0.14) cm·(sec·mmHg)^−1^, *p* = 0.03) (Figure 1, Panel D). Moreover, we observed a significant correlation between BMI and RVAC (r = −0.16, *p* = 0.05) (Figure 2). Importantly, even after adjustment for confounders (age, sex, systolic blood pressure, diastolic blood pressure, left ventricular ejection fraction, intraventricular thickness, left ventricular mass index, left atrial diameter index and the ratio of E/e’) in the multivariate regression analysis (Table 3), obesity remained associated with lower RVAC (β: −0.085, 95% confidence interval: −0.163, −0.009, *p* = 0.029).

## 4. Discussion

In this cohort study based on an echocardiographic registry, we found that subjects without known cardiovascular disease, evident left heart dysfunction or respiratory impairment present with a deterioration of RVAC according to BMI and their classification as overweight or obese. Regarding the indices of LV systolic function, we found no differences in deformation parameters and LV GLS according to obesity, although a clinical meaningless lower LVEF was found in obese subjects.

RVAC stands as a measure of the combined efficacy of the right ventricle to forward blood to the pulmonary circulation and of the pulmonary circulation to receive the right ventricular forward flow. The correlation between obesity and right heart failure is a multifaceted interplay involving intricate physiological mechanisms, clinical manifestations, and therapeutic considerations [3]. The prognostic role of RVAC has lately been evaluated in different settings, in patients with significant mitral regurgitation and transcatheter edge-to-edge repair [18], in patients with pulmonary hypertension [19,20], or in patients with HFrEF [21]. However, the role of RVAC in subjects without evident cardiovascular disease is undetermined. Even though in our study PASP and parameters of right ventricular systolic function (TAPSE, SRV) did not differ among groups, there was a stepwise decrease in RVAC (SRV/PASP) with BMI increase, which was evident even after adjustment for confounders, highlighting a significant correlation between RVAC and BMI. This parameter is an early indicator of impaired right ventricular performance, which is thought to become affected earlier than other systolic function parameters. This correlation highlights the effect of obesity on adverse right ventricular remodeling and systolic function, and the importance of timely imaging in order to detect early subclinical indications of impaired right cardiac function in seemingly asymptomatic overweight or obese individuals without established cardiovascular disease.

Several mechanisms can be implicated in the observed RVAC impairment in obese subjects as the increase in circulatory volume and the chronic volume overload of the LV or the obstructive respiratory pattern in obese subjects, although our subjects were selected to be free of respiratory impairment and there was no evidence of volume overload [22,23]. Additionally, obesity is associated with low-grade systemic inflammation and several adipocytokines (tumor necrosis factor alpha, leptin, resistin, inteleukin-6, etc.) may be found elevated with a direct or indirect impact on the heart and pulmonary circulation [24,25]. Interestingly, adipose tissue deposition may also be found in the epicardium, pericardium and in the chest wall, and may act through paracrine or endocrine mechanisms [26]. Indeed, thickened epicardial adipose tissue has been found to be associated with RV systolic impairment in patients with diabetes mellitus [27].

Regarding the right ventricle structure, we found a stepwise increase in right ventricular dimensions, as represented by right ventricular basal diameter, according to BMI category. Overweight and obese subjects exhibited significantly larger right ventricular dimensions compared to those with normal weight. These findings provide a plausible explanation for impaired exercise capacity and symptoms that overweight and obese patients may experience, particularly during exertion or at the occurrence of other stresses on the right ventricle, such as an increase in pulmonary arterial pressure due to pulmonary embolic events or respiratory infections. Importantly, studies have shown that addressing obesity and losing weight via lifestyle modifications, pharmacological agents, or surgical procedures, can reverse the remodeling of the right ventricle and improve functional status [28]. Therefore, an early detection of impaired right ventricular function is important as it could further prompt obesity management, leading eventually to the modification of right ventricular reverse remodeling and the restoration of normal function.

A pattern of inverse association between BMI and LVEF was also evident based on our results, although the differences observed were clinically insignificant and, most importantly, LV GLS did not differ in our study population. Several studies have examined the same question and although the impact of obesity on LV mass and volumetric indices was consistent, findings concerning the impact of obesity on LV systolic performance differ among different studies [29,30]. Moreover, there are no conclusions on the mechanisms linking obesity with LV systolic performance, with hyperglycemia-induced intra- and extra-cellular glycation of proteins, oxidative impact, and inflammatory damage in myocardial cells as proposed mechanisms [31].

Regarding the pathophysiological mechanisms identified in obesity-mediated heart failure, it is known that adipose tissue dysfunction, chronic inflammation, and insulin resistance emerge as central contributors to right ventricular remodeling and dysfunction [32]. Hence, increasing focus has been given on the ways in which obesity affects the right ventricle, regarding alterations in dimension, remodeling, as well as systolic function [33]. Obesity-related right ventricular enlargement and dysfunction can significantly compromise cardiac output and lead to clinical manifestations of heart failure. Research has shown that the right ventricle undergoes structural and functional changes in response to the increased metabolic and hemodynamic demands imposed by excess body weight, which highlights the importance of addressing these alterations to prevent progression to heart failure. The limitations of current imaging and diagnostic modalities in obese patients deserve particular attention. Issues such as suboptimal image quality, limited acoustic windows due to excess adipose tissue, and altered hemodynamics in obesity pose significant challenges for the accurate assessment of cardiac structure and function. These limitations underscore the need to refine existing echocardiographic techniques or develop novel imaging modalities specifically designed for this population. Advanced imaging techniques such as cardiac magnetic resonance imaging (MRI) and three-dimensional echocardiography may offer improved visualization and measurement accuracy, but further research is needed to optimize their use in obese patients [34,35]. Regarding therapeutic considerations, while it is known that weight management is crucial in preventing and treating right heart failure in obese individuals, it is also important to explore therapeutic strategies addressing molecular pathways implicated in obesity-related cardiac dysfunction, such as targeting adipokines and using anti-inflammatory agents [8]. Ongoing research is needed to uncover novel therapeutic targets and optimize existing strategies for the management of right heart failure in the context of obesity [36].

While our study offers valuable insights into the relationship between obesity and right ventricular-arterial coupling (RVAC), it is important to acknowledge several limitations. Firstly, the design of our study does not allow to establish the causality between obesity and right ventricular function indices. The sample size of our study, while adequate for initial analysis, may not be sufficient to detect smaller effect sizes, to generalize findings across diverse populations or to adjust for confounders such as differences in treatment status.

## 5. Conclusions

Obesity is associated with the adverse structural remodeling of the right ventricle, impaired right ventricular systolic performance and RVAC in subjects with no evident cardiovascular or respiratory disease. These findings suggest the potential impact of obesity on right heart function, emphasizing the importance of managing body weight to prevent obesity-related cardiac dysfunction. Future research should further investigate the mechanisms underlying these associations and explore potential therapeutic interventions to mitigate the adverse cardiovascular effects of obesity.

## Figures and Tables

**Table 1:** Characteristics of the study population stratified by BMI categories.

	Total (N = 145)	Normal Weight (N = 58)	Overweight (N = 53)	Obese (N = 34)	*p*-Value
Age, years	60.3 (17.0)	57.7 (18.6)	61.6 (15.3)	62.9 (16.5)	0.29
Male sex, %	46.9	43.1	52.8	44.1	0.55
BMI, kg/m^2^	27.1 (5.4)	22.5 (2.1)	27.4 (1.4) *	34.6 (4.4) *^,†^	<0.001
Hypertension, %	57.0	50.0	63.6	59.3	0.49
Diabetes mellitus, %	17.0	5.4	10.0	40.7 *^,†^	<0.001
Dyslipidemia, %	36.2	29.7	37.5	44.0	0.51
SBP, mmHg	133 (14)	134 (15)	132 (13)	132 (11)	0.84
DBP, mmHg	73 (9)	73 (10)	73 (8)	73 (9)	0.94
Heart rate, bpm	73 (16)	76 (16)	70 (14)	73 (16)	0.17

BMI: body mass index, SBP: systolic blood pressure, DBP: diastolic blood pressure. * signifies statistically significant difference (*p* < 0.025) when compared to normal weight subjects. ^†^ signifies statistically significant difference (*p* < 0.025) when compared to overweight subjects.

**Table 2:** Echocardiographic findings left atrial and ventricular function in the study population stratified by BMI categories.

	Total (N = 145)	Normal Weight (N = 58)	Overweight (N = 53)	Obese (N = 34)	*p*-Value
LV EF, %	56.8 (3.4)	57.4 (3.4)	57.1 (3.5)	55.5 (3.3) *	0.03
LV GLS, %	−20.0 (3.5)	−20.1 (3.4)	−20.3 (3.5)	19.5 (4.0)	0.81
LVEDDi, mm/m^2^	24.3 (2.9)	25.7 (2.9)	24.2 (2.5) *	22.2 (2.2) *^,†^	<0.001
LVESDi, mm/m^2^	16.2 (2.4)	16.6 (2.6)	16.4 (2.5)	15.3 (1.9) *	0.03
IVS, mm	9.1 (1.4)	8.8 (1.5)	9.3 (1.3)	9.4 (1.2)	0.07
PW, mm	8.7 (1.2)	8.3 (1.2)	8.8 (1.2)	9.0 (1.1) *	0.017
LVMi, g/m^2^	74.0 (16.8)	72.8 (17.7)	76.2 (16.9)	72.5 (15.4)	0.49
LADi, mm/m^2^	19.9 (3.7)	20.8 (4.2)	20.0 (3.3)	18.2 (2.7) *	0.006
E/e’	8.5 (4.0)	7.6 (2.7)	8.6 (3.3)	9.7 (6.0)	0.056

LV EF: left ventricular ejection fraction, LV GLS: LV global longitudinal strain, LVEDDi: LV end-diastolic diameter indexed, LVESD: LV end-systolic diameter indexed, IVS: interventricular septum, PW: posterior wall, LVMi: Left Ventricular Mass Index, LADi: left atrium diameter indexed. * signifies statistically significant difference (*p* < 0.05) when compared to normal weight subjects. ^†^ signifies statistically significant difference (*p* < 0.05) when compared to overweight subjects.

**Table 3:** Regression analysis on the association between obesity and RVAC.

Parameters	Univariate Analysis		Multivariate Analysis	
	β Coefficient (95% CI)	*p*-Value	β Coefficient (95% CI)	*p*-Value
Age	−0.004 (−0.006, −0.002)	<0.001	−0.002 (−0.004, 0.000)	0.053
Male sex	−0.004 (−0.061, 0.053)	0.90	−0.020 (−0.082, 0.041)	0.51
Normal weight (ref)				
Overweight	−0.053 (−0.176, 0.011)	0.10	−0.045 (−0.107, 0.018)	0.16
Obese	−0.095 (−0.168, −0.023)	0.010	−0.085 (−0.163, −0.009)	0.029
Hypertension	−0.069 (−0.139, 0.001)	0.06		
Diabetes mellitus	−0.051 (−0.146, 0.043)	0.29		
Dyslipidemia	−0.052 (−0.127, 0.022)	0.17		
SBP	0.002 (0.000, 0.004)	0.03	0.001 (−0.001, 0.003)	0.34
DBP	0.005 (0.002, 0.009)	<0.001	0.004 (−0.002, 0.008)	0.005
Heart rate	0.001 (−0.001, 0.003)	0.22		
LV EF	0.019 (0.011, 0.026)	<0.001	0.008 (−0.001, 0.017)	0.09
LV GLS	0.002 (−0.009, 0.013)	0.71		
LVEDDi	0.001 (−0.010, 0.010)	0.95		
LVESDi	−0.006 (−0.018, 0.006)	0.33		
IVS	−0.028 (−0.048, −0.008)	0.006	0.007 (−0.025, 0.038)	0.68
PW	−0.021 (−0.045, 0.003)	0.08		
LVMi	−0.002 (−0.003, −0.001)	0.02	0.001 (−0.002, 0.003)	0.87
LADi	−0.014 (−0.022, −0.007)	<0.001	−0.004 (−0.016, 0.008)	0.52
E/e’	−0.016 (−0.023, −0.009)	<0.001	−0.009 (−0.017, −0.001)	0.026
RVD1_basal_	−0.006 (−0.015, 0.003)	0.18		

SBP: systolic blood pressure, DBP: diastolic blood pressure, LV EF: left ventricular ejection fraction, LV GLS: LV global longitudinal strain, LVEDDi: LV end-diastolic diameter indexed, LVESD: LV end-systolic diameter indexed, IVS: interventricular septum, PW: posterior wall, LADi: left atrium diameter indexed, RVD1: right ventricular basal diameter at end-diastole.

## Data Availability

The raw data supporting the conclusions of this article will be made available by the authors on request.

## Supplementary Materials

The following supporting information can be downloaded at: https://www.mdpi.com/article/10.3390/jcm13123389/s1, Table S1: Echocardiographic findings of RV function and RVAC in the study population, stratified by BMI categories; File S1: STROBE Statement—Checklist of items that should be included in reports of cross-sectional studies.
