# Supplementary material for: Impairment in Right Ventricular-Pulmonary Arterial Coupling in Overweight and Obesity
Source: J Clin Med. 2024 Jun 10;13(12):3389. doi: 10.3390/jcm13123389 (PMC11203835; doi:10.3390/jcm13123389)
Supplement: Supplementary file 1 [file jcm-13-03389-s001.zip › Supplementary Table 1.pdf]

Supplementary Table 1. Echocardiographic findings of RV function and RVAC in the study population, stratified by BMI categories.					
	**Total (N=145)**	**Normal weight (N=58)**	**Overweight (N=53)**	**Obese (N=34)**	**p-value**
$RVD1_{basal}$, mm	32.6 (4.1)	31.6 (4.1)	33.1 (4.3)	34.0 (3.5)	0.053
TAPSE, mm	22.2 (3.3)	22.6 (3.4)	22.4 (3.5)	21.2 (2.6)	0.14
SRV, cm/sec	13.2 (2.7)	13.5 (2.8)	13.2 (2.3)	12.7 (2.9)	0.38
PASP, mmHg	30 (9)	28 (8)	30 (8)	32 (9)	0.14
RVAC, $cm\cdot(sec\cdot mmHg)^{-1}$	0.48 (0.17)	0.52 (0.19)	0.47 (0.16)	0.43 (0.14)*	0.03

RV: right ventricle, RVAC: right ventriculoarterial coupling, RVD1: Right ventricular basal diameter at end-diastole, TAPSE: Tricuspid annular plane systolic excursion, SRV: Peak systolic velocity of the tricuspid annulus, PASP: pulmonary artery systolic pressure

* signifies statistically significant difference ($p<0.05$) when compared to Normal weight subjects
